# Parents' Experiences and Perspectives Toward Tuberculosis Treatment Success Among Children in Malaysia: A Qualitative Study

**DOI:** 10.3389/fpubh.2020.577407

**Published:** 2020-12-15

**Authors:** S. Maria Awaluddin, Nurhuda Ismail, Siti Munira Yasin, Yuslina Zakaria, Norzila Mohamed Zainudin, Faridah Kusnin, Mas Ahmad Sherzkawee Mohd Yusoff, Asmah Razali

**Affiliations:** ^1^Department of Public Health Medicine, Faculty of Medicine, Universiti Teknologi MARA, Puncak Alam, Malaysia; ^2^Institute for Public Health, National Institutes of Health, Ministry of Health Malaysia, Shah Alam, Malaysia; ^3^Department of Pharmaceutical Life Sciences, Faculty of Pharmacy, Universiti Teknologi MARA, Puncak Alam, Malaysia; ^4^Sunway Medical Centre, Subang Jaya, Malaysia; ^5^Klang District Health Office, Selangor Health State Department, Ministry of Health Malaysia, Putrajaya, Malaysia; ^6^TB Unit, Selangor Health State Department, Ministry of Health Malaysia, Putrajaya, Malaysia; ^7^Sector of TB/Leprosy, Disease Control Division, Ministry of Health Malaysia, Putrajaya, Malaysia

**Keywords:** children, tuberculosis, qualitative, treatment success, parents' perspectives

## Abstract

**Introduction:** The trends of tuberculosis (TB) treatment success rate among children in Malaysia plateaued at 90% from 2014 to 2017. Malaysia sets a higher treatment success target of 95% to be achieved in line with an affordable, accessible, and holistic approach in managing TB among children.

**Objective:** This study aims to explore the parents' experiences and perspectives toward achieving treatment success among children who were diagnosed with TB in two districts in Selangor state, Malaysia.

**Methods:** The study was conducted using phenomenology study design via an in-depth interview of 15 mothers who were purposively sampled from the list of pediatric TB cases in the MyTB version 2.1 database in Klang and Petaling Districts of Selangor state. The R-based qualitative data analysis package of R version 0.2-8 was used to perform the thematic analysis.

**Results:** Two main themes were identified from this study. The first theme was trust toward the healthcare services with the subthemes of acceptance, self-efficacy, holistic care, and perceived benefits. The second theme was the motivation to take or continue medication. The subthemes were support from family, healthcare workers' (HCWs') support, the convenience of healthcare services, community support, personal strength, and child's character.

**Conclusion:** TB treatment success for children can be achieved when parents develop trust in healthcare services and have strong motivational factors to remain steadfast in achieving a successful treatment goal. Psychosocial support should be provided to the primary caregiver who faced any difficulty, while good relationships between parents and HCWs should be maintained. These results will inform the TB program managers to strengthen the holistic approach and identify the motivational factors among parents of children with TB disease.

## Introduction

Tuberculosis (TB) among children is estimated at about 11% of the total cases worldwide, which affects 1.1 million children aged 0–14 years in 2018 (1). TB among children is underdiagnosed and undertreated as the case detection gap is higher among children aged 0–4 years and those aged 5–14 years, 69 and 40%, respectively, than the case detection gap for all ages, which is 35% (2). The challenge in detecting TB among children is the difficulty to diagnose TB disease accurately due to its nonspecific symptoms and paucibacillary nature (3–6). Thus, TB among children requires strong public health attention because it shows recent infection in the community and unoptimized TB control activity (7, 8). In addition, children younger than 5 years have a higher mortality rate if they were not given appropriate TB treatment promptly (9).

TB is a curable disease, and the global treatment success rate is targeted at 90% (1). Malaysia has recorded treatment success rate of 90.4, 91.4, 91.4, and 90.1% from 2014 to 2017 for children with TB disease (10). Although Malaysia has achieved the targeted treatment success rate for children, it remained at a plateau trend, making the local treatment success goal of 95% seem unachievable (11). Parents' positive perception of TB treatment is associated with treatment success in children because they play essential roles in decision making for their children's health (12–15). The World Health Organization recommended a holistic approach in managing TB among children by including support from parents or family members to ensure better treatment adherence and success (16, 17). Strategies suggested to increase TB treatment adherence include providing education on TB to the family and community members, psychosocial support, financial support, and improving TB care delivery and health systems (18).

Previous exploratory studies had examined on the barriers and challenges among parents or caregivers who were dealing with a child with TB disease such as delay in diagnosis, difficulty in administering TB medication, inadequate TB education, and seeking traditional help before taking modern medicine (19, 20). Moreover, parents might experience psychological distress due to the burden arising from TB illness itself and unnecessary worry about the disruption of their children's education progress (13). Another study highlighted that parents had to make some adjustments in their time management as TB follow-up affected their working hours (21). Various studies have documented on the lack of awareness and TB knowledge among parents or caregivers of children with TB disease (20–23).

Parents' experiences and perceptions regarding TB disease and treatment in Malaysia are unexplored and undocumented. Understanding parents' experiences and perspectives toward attaining a successful TB treatment may encourage other parents to follow the same pathway in achieving a complete and successful treatment. This study aims at exploring the parents' experiences and perspectives toward successful TB treatment among children in two districts in Selangor state, Malaysia.

## Materials and Methods

### Setting and Participants

The study used a qualitative approach with phenomenological design, conducted from January to February 2020 in Klang and Petaling Districts in Selangor, the most urbanized state in Malaysia with 6.5 million population (24). Participants for this study were approached via the available contact numbers registered with the children database of MyTB v2.1, a national TB surveillance database with the assistance from staff working in the TB unit, Selangor State Health Department. The selected TB patients among children had to be a child who either had achieved treatment success or was continuing TB treatment for the registration year of 2017 until 2019. The participants must be either the mother, father, or guardian 18 years or older who were taking care of the child during the TB illness and TB treatment phase. The sample size was estimated using maximum variation sampling technique to ensure that the data were collected from participants with a broad range of sociodemographic characteristics. A total of 12 participants were planned to be interviewed after taking into consideration the participants' socioeconomic status and children's age as suggested by previous literature (25, 26).

### Data Collection

In-depth interviews (IDIs) were conducted using a topic-based protocol exploring participants' experiences and their perspectives from the start of health changes until treatment completion. The main topics and questions are shown in Table 1. The interview probing technique was applied during the face-to-face interview session by a trained researcher at home settings. Some of the interviews were done in restaurants as per participants' request. The interviews were performed in Malay language after obtaining participant's informed written consent with an average duration of 40 min−1 h. Participants were assigned a unique identification number to ensure anonymity. Data saturation was taken into account and to be achieved when there were no new themes arose from the recent IDI sessions (27). The sessions were recorded, supplemented by the researcher's note-taking. The audio files were transcribed verbatim in Malay language. Confidentiality was ensured throughout the interview process and transcribing the audio files, with the researcher alone having access to the data.

**Table 1:** Sample of interview questions.

**Topic of interview**	**Main questions**
Detecting health changes and TB disease confirmation	1. What are the first health changes of your child at the initial phase?
	2. How was your child being detected as having TB disease?
TB treatment phase	3. Can you tell me how was your child when he/she underwent the TB treatment?
	4. What are the challenges to complete the TB medication?
Perceived benefits and supports	5. How was your child condition after TB treatment?
	6. What kinds of support that you received during TB treatment of your child?
Response from community	7. How was the neighborhood reaction after your child being diagnosed as having TB?
	8. How was the school acceptance toward your child?

### Data Analysis

Thematic analysis was conducted using R-based qualitative data analysis package of R version 0.2-8 (28). The selected participants' quotes were highlighted, and a suitable coding was assigned deductively and inductively. The validity of data was ensured through triangulation of participants' quotes, member checking, peer debriefing, and reflective note. The study procedures were documented to allow reproducibility (25). The transcription notes were checked repeatedly with the audio files to minimize apparent errors. The QOREQ-32 item checklist was used for reporting of this study (29).

### Ethical Approval

This study was authorized by the Research Ethics Committee (REC), UiTM, and the Medical Research and Ethics Committee (MREC), Ministry of Health Malaysia with registration number NMRR-19-1201-47959.

## Results

Of the 43 selected contact numbers for registered children with TB disease contacted using the “WhatsApp” application, only 15 participants agreed to participate and successfully interviewed. The sociodemographic characteristics of the participants, age and TB diagnosis of the child, are tabulated, as shown in Table 2. All participants were of Malay ethnicity and the mother of the child.

**Table 2:** The sociodemographic of the participants for the study on parents' perspectives toward TB treatment success among children with TB in Klang and Petaling districts; *N* = 15.

**Characteristics of the participants and**	**Number of participants**
**their child**	***n* (%)**
**Age of participants, y**	
20–29	3 (20.0)
30–39	6 (40.0)
40–49	4 (26.7)
50–59	2 (13.3)
**Education status**	
Tertiary	7 (46.7)
Secondary	8 (53.3)
**Age of child based on MyTB v2.1 Database**	
1–4 years	7 (46.7)
5–14 years	8 (53.3)
**Type of TB**	
Extrapulmonary Tuberculosis (EPTB)	3 (20.0)
Pulmonary Tuberculosis (PTB)	12 (80.0)
**Treatment outcome**	
Success	12 (80.0)
Still on treatment	3 (20.0)

Two main themes were identified from this study, exploring parents' experiences and perspectives toward achieving TB treatment success for their child. The themes were trust in the healthcare services and motivation to take or continue treatment for their child, as shown in Table 3.

**Table 3:** Summary of the thematic analysis of parents' perspectives toward TB treatment success among children with TB in Klang and Petaling districts.

**Themes**	**Subthemes**
Trust in the health care services	•Acceptance •Self-efficacy •Holistic care •Perceived benefits
Motivation to take or continue treatment for her child	•Support from family •HCWs support •Convenience healthcare services •Community support •Personal strength •Child character

### Theme 1: Trust in the Healthcare Services

Trust in the healthcare services consisted of four subthemes: acceptance, self-efficacy, holistic care, and perceived benefits.

#### Acceptance

Acceptance means the participants accepted the child's illness and complied with the required treatment. Participants sought medical help as soon as they detected health changes of their child, although it started with mild illness, e.g., common cough symptoms. As the problem worsened, one participant visited the emergency department, whereas other participants sought a second opinion in different health facilities.

“Initially, I gave medication for his fever; it resolved but was recurrent for about one month until one night he had a spiking fever. I went straight to the emergency department, and he was admitted.”

Participants accepted the facts that her child was having a serious illness and needed treatment after a thorough explanation by the doctor.

“I asked the doctors in the hospital ward, whether my daughter has TB disease. They confirmed it as they said my daughter had already given TB medication for three days. [] I just accepted if my daughter had TB disease, and I will give her the medication.”

A participant who had the previous history of other family members with TB disease ensured that her child could achieve treatment success.

“I think that those who have been infected with TB disease, the only way to cure the disease is by taking medication as instructed and remaining self-disciplined throughout the treatment phase.”

#### Self-Efficacy and Self-Empowerment

Parents tried to adapt themselves after discovering that their child was diagnosed as having TB disease. They readjusted their life in terms of psychological mindset, time management, and career commitment throughout this phase. They set priority to giving medications to their child despite traveling to their hometown or going for short vacations.

“*My mother and I took turns to accompany my ill daughter in the hospital. I went to work in the morning and looked after her in the evening. […] My advice for those who will be going through this similar situation, you must remain calm and do not overstress yourself. If we are having low mood and are always under pressure, we cannot help our child. We must be a strong mother. Just listen carefully to the doctor and follow their advice as the doctor said TB could be cured. […] I will bring her medication every time we were away from home, and she definitely will be given the TB medication on time.”*

Participants adjusted their timetable to meet the TB appointment as scheduled, not to miss schooling.

“The clinic appointment was in the evening session. I waited for my son at school around one o'clock, and we headed straight to the hospital. I took time off from my work for that evening session.”

Participants had high belief in themselves to become the DOT (directly observed treatment) supervisors for their child. They made sure that the amount of medication was adequate for the total number of days before the next scheduled appointment. Participants observed their child taking the pill themselves on a timely basis.

“After completing two weeks of DOT at health facility, I continued it at home and signed the medication record book. I observed her every day at 8.30 a.m. sharp. [] I also asked about her urine color to make sure everything was consistent.”

They applied many techniques to ensure their child took the medication every day.

“I needed to persuade him on taking medicine a day before. I will talk with him nicely and sometimes offer ice cream as a reward. His moods need to be handled carefully, and I need to keep my emotion in check.”

#### Holistic Care

Holistic care refers to management of TB diseases from the early case detection, prompt treatment, continuous counseling, and posttreatment follow-up. Four participants mentioned that their child was detected through an active case detection program because her child had an exposure with a sputum-positive Pulmonary Tuberculosis (PTB) adult patient. The healthcare team visited the participants' house and the child's school to provide education on TB disease. Participants mentioned that they were counseled on TB medication, disease prevention, and personal hygiene by the healthcare workers (HCWs), prior to discharge from the hospital.

#### Perceived Benefits

Participants observed their child's health status closely and noted the changes such as improvement in their child's appetite and weight gain after taking TB medication. They noticed that their child resumed their activities prior to falling ill. Participants agreed that they gained new information regarding TB disease, which had benefited them extensively.

### Theme 2: Motivation to Take or Continue Treatment for Her Child

The motivation to seek help during the initial phase and to continue the TB treatment came from family support, HCWs' support, convenient healthcare services, community support, personal strength and child's character.

#### Support From Family

Motivation can be provided from the family members themselves, when participants received necessary supports from their husbands and their extended families.

“My mother helped me in taking care of the youngest while I was in hospital. I also got support from my siblings in terms of finances and transportation.”

Participants mentioned that their husbands shared responsibilities such as taking care of their child in the hospital ward, giving TB medication, and sending them to the hospital for an appointment.

#### HCWs' Support

HCWs play a vital role in giving assurance to the participants regarding the chances of curing the disease, alleviating unnecessary worries, and continuously providing health education during TB follow-up.

“The way of explanation and assurance given by the doctor soothed my emotion. I felt a bit relieved and I accepted the fact that my daughter has TB disease and needs treatment.”

#### Convenience of Healthcare Services

The convenient amenities and efficient services provided by the healthcare facilities have also eased the participants to achieve treatment success for their child. For instance, a patient-centered approach was implemented whereby participants experienced the flexibility of collecting TB medication.

“If we have a problem to collect the medication as per scheduled appointment, we just inform them (staff of the clinic) earlier. They can help us, and it is quite flexible, but do not miss the medication.”

Some participants received support material such as face masks and TB education pamphlets.

“The clinic's staff supplied me with face masks and some vitamins for increasing appetite.”

#### Community Support

Community support may positively encourage participants to remain steadfast during the TB treatment phase of their child. It includes workplace support, where participants can avail of longer and frequent annual leave.

“My employer was good. I use my annual leave as I had a lot of it. He allowed me to settle my daughter's health issues first.”

Supportive communication with neighbors and friends as well as during a chat in social media also motivates the participants.

“I made a post in my social media account. A friend commented and shared her previous experiences, basically the same thing to solve this problem, by taking the medication.”

Participants experienced a greater understanding from schoolteachers regarding their child's illness.

“The schoolteacher gave their cooperation and support. My son and his friends continued to eat together from the same food tray.”

#### Personal Strength

Participants were self-confident to face any hardship in life. Two of the mothers were pregnant, whereas another participant was also an adult TB patient.

“I took care of my child during my eight months of pregnancy. I must be strong psychologically.”

“I was already on TB medication for myself. I put on weight and was feeling energetic to take care of her.”

#### Child's Character

Participants confessed that the reason for success might be contributed by their child themselves being obedient and remaining active despite their illness. The child's progress became a motivation to the mother that TB medication worked.

“She listened to my words”

“During the illness phase, I never saw my child look ill and weak. He experienced mild fever but looked active and energetic.”

## Discussion

This study explores the parents' experiences and perspectives toward achieving successful TB treatment among mothers who were the primary caregivers of a child with TB disease among the selected TB patients in this study, 12 of 15 children had achieved treatment success. The main identified themes included trust in healthcare services and the presence of motivation to take or continue TB medication. Previous studies explored the challenges and barriers faced by caregivers or parents of children with TB as the primary focus (20, 21).

Participants experienced trust in the healthcare system as pictured by four subthemes of acceptance, self-efficacy, holistic care, and perceived benefits. The participant's behavior of seeking appropriate medical assistance after realizing that their child was ill explained their acceptance. The acceptance might be influenced by the accessibility and affordability of the healthcare service provided in the urbanized Selangor state. Our study observed that all the children in this study received treatment in a public hospital, whereas different preferences were observed in another study (30). In terms of medical and alternative treatment choices, this finding might differ if the research was conducted in rural areas or in the place where alternative medicine was given more priority than medical treatment (19). The trust in TB medication forced participants to face the daily challenges of administering the medication. These parents gained knowledge of TB disease, which strengthened their faith in the healthcare services offered. Various previous studies supported on the lack of TB knowledge among caregivers and patients that may result in a poor outcome (13, 23, 31). Besides that, seeking alternative medicine or over-the-counter medicine due to mild symptoms and any distrust in the public healthcare system may complicate and delay the appropriate treatment (32–35).

This study observed that TB disease had affected the participants' daily routine such as the need to stay in hospital wards and fulfill the child's need during illness, and some participants even needed to take long leave from work. These problems are similar to previous studies (13, 20, 21). Parents in this study felt that they managed to adapt with the problematic situation of taking care of a sick child because they had self-efficacy, a belief on the ability to complete the required action following a problem (36). Participants faced many challenges such as time management, transportation problem, personal conflicts, administration of medication challenges, and financial constraints, but they finally achieved treatment success for their child as they had a higher level of self-efficacy (21, 37).

The holistic care in combating TB disease was associated with successful treatment that starts from early case detection and treatment, continuous clinic follow-up, and community health follow-up (30). The presence of multiple clinic follow-ups from the primary care to the tertiary level involving multidisciplinary specialist teams results in satisfaction of the quality of care delivered to TB patients. In addition, counseling session on medication adherence before hospital discharge played a vital role to enhance caregiver understanding of TB medication and promote treatment adherence at home (38).

Perceived benefits among participants strengthened the trust in the existing healthcare system. The benefits can be observed through health status improvement of the child after taking TB treatment. Children can regain their previous healthy status even with a short duration of TB medication; thus, it is important to ensure the completion of total period of TB treatment by continuous DOT and clinic follow-up. Parents of children in this study who have completed TB treatment confessed that they strictly adhered to the HCW's advice because of fear of restarting the drug regimen and fear of TB complications such as death (21, 30).

The second theme explained the support received by the participants, which acted as the motivation for parents in taking or continuing TB treatment for their child. Support from the family members might come from the spouse, siblings, or grandparents of the child, such as taking turns in looking after the sick child, giving medication, or even taking care of other children in the family when the participants stayed in the hospital ward. Strong family support is needed to motivate the primary caregivers including the history of successful TB treatment among family members as observed in previous studies (20, 21, 37).

HCWs performed an essential role in giving the correct information, a calm but reassuring explanation on TB disease, its complication, and plan of treatment. Various studies observed that a mutual relationship between caregiver and HCWs increased the treatment adherence of their patients (21, 30, 37). This study also found a similar relationship between caregiver and HCWs in terms of correct information, positive advice, and effective communication. The positive relationship boosts the confidence level of parents in achieving curable status for their child by adhering to TB medication (30).

The participants described convenient healthcare services through the presence of flexibility in terms of appointment date for follow-up and medication. Participants needed some adjustments in their life to meet the scheduled appointment, and flexibility of the services motivated them to adhere to their child's TB treatment. The flexibility of the services was also mentioned in a previous study (21).

Support from the community was related to the workplace of the participants, and persons who had a close relationship with the children such as teachers, friends, neighbors, and newly made friends in the hospital ward. Encouragement from employers and colleagues at work had bolstered their self-abilities and hence able to provide more attention to their child's health status. For working mothers, on top of their husband's and extended family's support, the employer's support was equally important. Excellent support from employer denoted that the working mothers were given flexibility in taking annual leave, and the employers understood the psychological strain faced by their employee and tried to ease the burden.

Participants in this study and other studies received supportive comments from their newly made friends in the hospital ward who shared their similar previous experience in dealing with TB disease (37). The decision to seek medical help was influenced by persons who were close with the child, such as teachers who made an observation of the child in school (13). It may also assist parents in making the right decision for an early treatment. This study also observed that children with TB disease were not discriminated in terms of attending school, playing with friends, and interacting with teachers after the child returned to school. Another important cue of action was the news regarding death due to TB disease in the community. Because of fear of fatal complications, they worked hard to ensure treatment adherence of their child (21).

Participants in this study exhibited personal strength in terms of their self-confidence. The commitment given toward completing TB treatment and compliance was shown by the mothers, although few mothers confessed that they required help from their husbands and their mothers to administer TB medication. A similar reaction to the mother's commitment was reported in another study (21). The child's character was one of the factors that motivated these mothers. An obedient child with good discipline increased treatment adherence and treatment completion. For the younger age group, a physically active child gave rise to a positive outcome, thus allowing mothers to remain calm and compliant with the daily routine of administering TB medication to their child.

### Strengths and Limitations

This study was a primary research and had achieved richness of the data because a thorough exploration of the experiences was conducted in sequence from the initial phase of detecting health changes until TB treatment completion. Furthermore, the interviewer had developed a good rapport with the participants, which allowed an in-depth probing during the interview sessions. A few limitations were identified in this study. First, the participants were only among mothers; therefore, fathers' or other guardians' perspectives were not explored. Second, the participants were all of Malay ethnicity, in which parents' views of different ethnicities were not known. Besides that, the differences in parent's perspectives according to urban or rural residence cannot be identified.

## Conclusion

TB treatment success for children can be achieved when parents develop trust in healthcare services and have strong motivational factors to remain steadfast in achieving a successful treatment goal. Psychosocial support should be provided to primary caregivers who face any difficulty, and a good relationship between parents and HCWs should be maintained. These results will inform the TB program managers to strengthen the holistic approach and identify the motivational factors among parents of children with TB disease to promote treatment adherence and finally their successful treatment.

## Data Availability Statement

The raw data supporting the conclusions of this article will be made available by the authors, without undue reservation.

## Ethics Statement

The studies involving human participants were reviewed and approved by 1. The Research Ethics Committee (REC) of Universiti Teknologi Mara (UiTM) 2. Medical Research and Ethics Committee (MREC), Ministry of Health of Malaysia (MOH). The patients/participants provided their written informed consent to participate in this study.

## Author Contributions

SA, NI, SY, YZ, NM, and FK contributed in designing the study, data cleaning, data analysis, interpretation of the findings, and drafted the manuscript. SA conducted IDI and transcription. The paper has been reviewed and critiqued by MM and AR. All authors contributed to the article and approved the submitted version.

## Conflict of Interest

The authors declare that the research was conducted in the absence of any commercial or financial relationships that could be construed as a potential conflict of interest.
