# Supplementary material for: Parents' Experiences and Perspectives Toward Tuberculosis Treatment Success Among Children in Malaysia: A Qualitative Study
Source: Front Public Health. 2020 Dec 15;8:577407. doi: 10.3389/fpubh.2020.577407 (PMC7770179; doi:10.3389/fpubh.2020.577407)
Supplement: Supplementary file 1 [file Table_1.docx]

Supplementary Material

**Manuscript title:** Parents’ Experiences and Perspectives Towards TB Treatment Success among Children in Malaysia: A Qualitative Study

**Appendix 1**

**Consolidated criteria for reporting qualitative studies (COREQ): 32-item checklist**

Developed from:

Tong A, Sainsbury P, Craig J. Consolidated criteria for reporting qualitative research (COREQ): a 32-item checklist for interviews and focus groups. *International Journal for Quality in Health Care*. 2007. Volume 19, Number 6: pp. 349 – 357

| **No. Item** | **Guide questions/description** | **Reported on Page #** |
| --- | --- | --- |
| **Domain 1: Research team and reﬂexivity** |  |  |
| *Personal Characteristics* |  |  |
| 1. Interviewer/facilitator | Which author/s conducted the interview or focus group? | SMA conducted IDI for 15 participants |
| 2. Credentials | What were the researcher’s credentials? E.g. PhD, MD | MD |
| 3. Occupation | What was their occupation at the time of the study? | She works as a researcher at the National Institutes of Health, Centre for Epidemiological Study. During this study, she was a student for Master of Science (Medicine) |
| 4. Gender | Was the researcher male or female? | Female |
| 5. Experience and training | What experience or training did the researcher have? | Joined training for research methodology, qualitative study during her period of MSc study |
| *Relationship with participants* |  |  |
| 6. Relationship established | Was a relationship established prior to study commencement? | Yes  . |
| 7. Participant knowledge of the interviewer | What did the participants know about the researcher? e.g. personal goals, reasons for doing the research | An interview protocol and participant’s information sheet were given to participant as well as a verbal explanation prior to the interview. |
| 8. Interviewer characteristics | What characteristics were reported about the inter viewer/facilitator? e.g. Bias, assumptions, reasons and interests in the research topic | My reflexivity: “I conducted this study with my background as a medical doctor for 18 years and had previously contracted TB disease from my husband five years ago. I was diagnosed with TB disease slightly later and had completed six months of treatment duration. I experienced the situation of being a TB patient myself and also as a caregiver to my children, and had faced multiple challenges in completing TB treatment.” |

| **Domain 2: study design** |  |  |
| --- | --- | --- |
| *Theoretical framework* |  |  |
| 9. Methodological orientation and Theory | What methodological orientation was stated to underpin the study? e.g. grounded theory, discourse analysis, ethnography, phenomenology, content analysis | Phenomenology design,  Topic-based according to Health Belief Model theory constructs |
| *Participant selection* |  |  |
| 10. Sampling | How were participants selected? e.g. purposive, convenience, consecutive, snowball | Criterion sampling  The aim was looking for parents of children with TB disease who were the primary caregiver, aged 18 years and above and has a child either has completed TB treatment or still taking the medication. |
| 11. Method of approach | How were participants approached? e.g. face-to-face, telephone, mail, email | Contacted via “WhatsApp’s” application, followed by a face-to-face interview |
| 12. Sample size | How many participants were in the study? | After 15 participants, and no new codes or themes were detected, data has achieved its saturation level; hence interview was stopped. |
| 13. Non-participation | How many people refused to participate or dropped out? Reasons? | Out of 43 contact numbers being contacted, only 15 participants responded and successfully interviewed. Seventeen participants responded for one time and 11 were non- response at all.  Reasons: refuse to respond |
| *Setting* |  |  |
| 14. Setting of data collection | Where was the data collected? e.g. home, clinic, workplace | Home setting: 10 participants  Restaurants: 3 participants.  Workplace: 2 participants. |
| 15. Presence of non-participants | Was anyone else present besides the participants and researchers? | Yes, participants companion such as her children, husband, in-laws |
| 16. Description of sample | What are the important characteristics of the sample? e.g. demographic data, date | The primary caregiver aged 18 years and above and has a child who has been diagnosed with TB disease either has completed TB treatment or still taking the medication. |
| *Data collection* |  |  |
| 17. Interview guide | Were questions, prompts, guides provided by the authors? Was it pilot tested? | Provided as in Table 1 in the text file. The interview protocol was discussed with three panels of expert in TB disease, (TB stake holder, TB programme manager, clinician) |
| 18. Repeat interviews | Were repeat inter views carried out? If yes, how many? | No repeat interviews |
| 19. Audio/visual recording | Did the research use audio or visual recording to collect the data? | Audio files were recorded with a tape recorder |
| 20. Field notes | Were ﬁeld notes made during and/or after the interview or focus group? | Yes, conducted concurrently |
| 21. Duration | What was the duration of the inter views or focus group? | 40 minutes to one hour |
| 22. Data saturation | Was data saturation discussed? | Yes, as per explanation in item No. 12 |
| 23. Transcripts returned | Were transcripts returned to participants for comment and/or correction? | No. Not suitable for current situation as participants cannot be disturbed repeatedly. |
| **Domain 3: analysis and ﬁndings** |  |  |
| *Data analysis* |  |  |
| 24. Number of data coders | How many data coders coded the data? | Only one, SMA. |
| 25. Description of the coding tree | Did authors provide a description of the coding tree? | Codebook, subthemes and themes were available |
| 26. Derivation of themes | Were themes identiﬁed in advance or derived from the data? | Both, inductive and deductive |
| 27. Software | What software, if applicable, was used to manage the data? | R-based qualitative data analysis (RQDA) package of R version 0.2-8 |
| 28. Participant checking | Did participants provide feedback on the ﬁndings? | The summary for each interview was discussed with participants at the end of the interview session. |
| *Reporting* |  |  |
| 29. Quotations presented | Were participant quotations presented to illustrate the themes/ﬁndings? Was each quotation identiﬁed? e.g. participant number | Yes, as per result section |
| 30. Data and ﬁndings consistent | Was there consistency between the data presented and the ﬁndings? | Yes, there was. |
| 31. Clarity of major themes | Were major themes clearly presented in the ﬁndings? | Yes. they were. |
| 32. Clarity of minor themes | Is there a description of diverse cases or discussion of minor themes? | Discussion of major and minor themes were conducted. |
